# Effectiveness of extracorporeal shock wave for low back pain: A protocol of systematic review

**DOI:** 10.1097/MD.0000000000014511

**Published:** 2019-02-15

**Authors:** Wei Wei, Hua-yu Tang, Yu-zhi Li, Tian-shu Wang

**Affiliations:** a Second Ward of Orthopedis Department; b Department of Urology, First Affiliated Hospital of Jiamusi University, Jiamusi, China.

**Keywords:** effectiveness, extracorporeal shock wave, low back pain, safety, systematic review

## Abstract

**Background:**

Previous clinical trials have reported that extracorporeal shock wave (EPSW) can be used to treat low back pain (LBP), and have achieved satisfied effect. However, its effectiveness is still inconclusive. Thus, this systematic review will aim to assess the effectiveness and safety of EPSW for patients with LBP.

**Methods:**

In this systematic review, the electronic databases of Cochrane Central Register of Controlled Trials, EMBASE, PUBMED, Cumulative Index to Nursing and Allied Health Literature, China National Knowledge Infrastructure, Chinese Biomedical Literature Database, Chinese Scientific Journal Database, and Wanfang Data will be searched from inception to January 1, 2019. Randomized controlled trials and case-control studies that assessed the effectiveness and safety of EPSW for LBP will be included. The primary outcome is pain intensity. The secondary outcomes are functional status, quality of life, psychological outcomes, as well as the adverse events. All process of the study selection, data extraction, and methodology evaluation will be carried out by two authors independently. RevMan 5.3 software will be utilized for statistical analysis.

**Results:**

This study will provide a detailed summary of latest evidence related to the effectiveness and safety of EPSW in pain relief, improvement of functional status, quality of life, and psychological disorders in patients with LBP.

**Conclusion:**

The findings of this study may provide possible guidance for LBP treated by EPSW.

**Dissemination and ethics:**

Ethical approval is not required in this study, because it will not collect the original data from individual patient. The results are expected to publish through a peer-reviewed journal.

**Systematic review registration:**

PROSPERO CRD42019120501.

## 1 Introduction

Low back pain (LBP) is the second most common disorder that affects adult population.^[1–4]^ It has been reported that its prevalence rate is 84% in a lifetime for a general population.^[5,6]^ Many factors are reported to contribute to the LBP, such as nerves, bones, musculatures, fascia, joints, and any other conditions that may result in this disorder.^[7–10]^ If this condition cannot be treated effectively, it may result in very poor quality of life in patients who experienced LBP.^[11–13]^

Treatment approaches for this condition mainly include pharmacological and non-pharmacological treatments.^[14,15]^ However, pharmacological treatments often involve limited efficacy and most importantly bring a variety of adverse events for patients.^[16,17]^ Thus, current treatment guidelines for this condition mainly focus on the non-pharmacological treatments, as recommended by The Guidelines for Chronic Back Pain of the American College of Medicine, published in 2017.^[18]^

Extracorporeal shock wave (EPSW) is reported to treat LBP effectively with fewer adverse events.^[19,20]^ However, there is limited evidence concerning its effectiveness and safety of EPSW for patients with LBP. Thus, this protocol of systematic review aims to determine the effectiveness and safety of EPSW for LBP, providing a scientific evidence for clinical decision.

## 2 Methods and analysis

### 2.1 Eligibility criteria for study selection

#### 2.1.1 Types of study

Only randomized controlled trials (RCTs) and case-control studies assessing the effectiveness and safety of EPSW for LBP will be included. We will exclude any other studies, such as nonclinical trials, case reports, and case series.

#### 2.1.2 Types of participant

Patients with clinically diagnosed of LBP will be included without restrictions of race, sex, and age.

#### 2.1.3 Types of intervention

Any included studies for evaluating the effectiveness and safety of EPSW only for patients with LBP will be included. Additionally, the studies using the EPSW plus other therapies will be excluded. The control interventions can be the placebo, medications, and so on, except the EPSW.

#### 2.1.4 Types of outcome measurement

The primary outcome includes pain intensity. It can be assessed by the visual analogue scale or other pain score tools.

The secondary outcomes comprise functional status, quality of life, psychological outcomes, as well as the adverse events. The functional status can be measured by the Roland–Morris Disability Questionnaire or other related tools. The quality of life, and psychological outcomes can be examined by the 36-Item Short Form Health Survey and Beck Depression Inventory, respectively, or any other associated scales.

### 2.2 Literature search

We will search the databases of Cochrane Central Register of Controlled Trials (CENTRAL), EMBASE, PUBMED, Cumulative Index to Nursing and Allied Health Literature, China National Knowledge Infrastructure, Chinese Biomedical Literature Database, Chinese Scientific Journal Database, and Wanfang Data from inception to January 1, 2019 without language restrictions. Reference lists of included studies will also be checked to identify any potential eligible trials. The search strategy for CENTRAL is presented in Table 1. The similar detailed strategies will be used for literature search from other electronic databases.

**Table 1:**
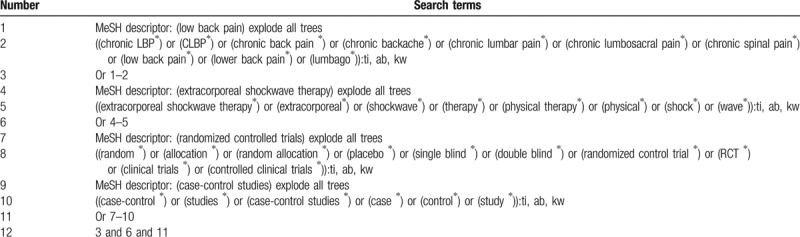
Search strategy applied in CENTRAL database.

### 2.3 Study selection

**Figure 1:**
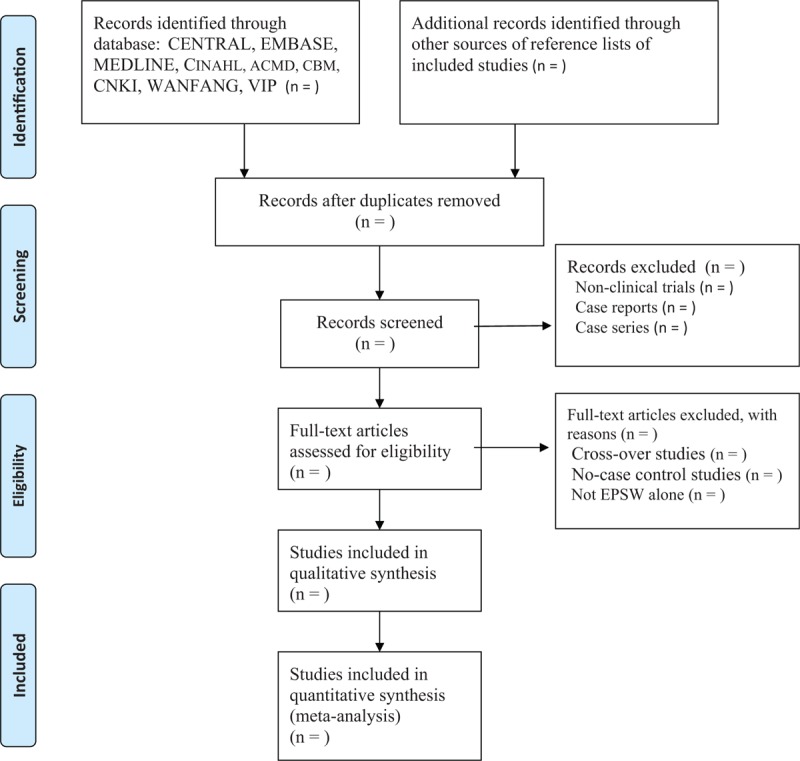
Diagram flow of study selection.

Two authors will independently check the titles, abstracts, and full texts, and will select all potential eligible studies against all inclusion and exclusion criteria. All disagreements will be settled down through discussion with a third author joined in. The study flow diagram is presented in Figure 1.

### 2.4 Data extraction and management

Data extraction will be independently performed by two authors using a predefined standardized data extraction sheet. The following information will be extracted: first author, published year, location, study design and methods, interventions, outcomes, and any other reporting information. A third author will be invited as an arbiter if divergences occur between two authors. We will contact the original authors to require the missing data if those data are not available. If those data are not obtainable, we will analyze the present available data.

### 2.5 Risk of bias assessment

Risk of bias assessment will be evaluated by using the criteria as described in details of the Cochrane Handbook of Systematic Review of Interventions. Two authors will independently evaluate each included study, with a third author acting as an arbiter through discussion if any differences regarding the risk of bias assessment arise.

### 2.6 Statistical analysis

#### 2.6.1 Treatment effect measurement

We will express the treatment effects using risk ratio with 95% confidence intervals (CIs) for dichotomous data, and mean difference or standardized mean difference with 95% CIs for continuous data.

#### 2.6.2 Assessment of heterogeneity

We will check the heterogeneity using the test of *I*^2^ and *χ*^2^. The heterogeneity is considered as acceptable if *I*^2^ ≤ 50%. Otherwise, significant heterogeneity is considered, and subgroup analysis should be conducted after the data pooled.

#### 2.6.3 Data synthesis

Outcome data will be pooled by using fixed-effect model with acceptable heterogeneity. Otherwise, we will use random-effect model to pool the data, and we will also perform subgroup analysis. We will not consider pooling the data if the heterogeneity is still substantial after the subgroup analysis.

#### 2.6.4 Subgroup analysis

We will conduct the subgroup analysis when the heterogeneity is not acceptable. It will be performed according to the different treatments, controls, outcome tools.

#### 2.6.5 Sensitivity analysis

We will carry out the sensitivity analysis to check the robustness of the pooled results. It will be conducted depending on the different methodological qualities, and statistical models.

#### 2.6.6 Publication bias

We will conduct the funnel plot to check the publication bias when more than 10 eligible trials are included. Meanwhile, we will also carry out Egger's and Begg's tests to check asymmetry of funnel plot.

## 3 Discussion

To our best knowledge, this systematic review firstly explores the effectiveness and safety of EPSW for LBP. It will supply a detailed summary of the current evidence relevant of EPSW in pain relief, improving functional status, quality of life, as well as the psychological outcomes of patients with LBP. This evidence may be helpful to the clinical practice, patients, and health policy makers regarding the use of EPSW in the treatment of LBP.

## Author contributions

**Conceptualization:** Wei Wei, Tian-shu Wang.

**Data curation:** Wei Wei, Hua-yu Tang, Yu-zhi Li, Tian-shu Wang.

**Formal analysis:** Wei Wei, Hua-yu Tang, Tian-shu Wang.

**Funding acquisition:** Tian-shu Wang.

**Investigation:** Yu-zhi Li, Tian-shu Wang.

**Methodology:** Wei Wei, Hua-yu Tang, Yu-zhi Li.

**Project administration:** Tian-shu Wang.

**Resources:** Wei Wei, Hua-yu Tang.

**Software:** Wei Wei, Hua-yu Tang, Tian-shu Wang.

**Supervision:** Hua-yu Tang, Yu-zhi Li.

**Validation:** Wei Wei, Yu-zhi Li, Tian-shu Wang.

**Visualization:** Yu-zhi Li, Tian-shu Wang.

**Writing – original draft:** Wei Wei, Yu-zhi Li, Tian-shu Wang.

**Writing – review & editing:** Wei Wei, Hua-yu Tang, Tian-shu Wang.
